# Single‐cell profiling guided combination therapy of c‐Fos and histone deacetylase inhibitors in diffuse large B‐cell lymphoma

**DOI:** 10.1002/ctm2.858

**Published:** 2022-05-23

**Authors:** Oliver H. Krämer, Günter Schneider

**Affiliations:** ^1^ Department of Toxicology University of Mainz Medical Center Mainz Germany; ^2^ Department of General, Visceral and Pediatric Surgery University Medical Center Göttingen Göttingen Germany

**Keywords:** acetylation, c‐FOS, DNA damage, HDAC, HDACi, salvage therapy

## Abstract

In this commentary on Wang, Wu, Xia, and colleagues, *Clinical Translational Medicine*, 2022, we sum up and discuss recent evidence on the regulation and relevance of the transcription factor c‐FOS in diffuse large B cell lymphoma cells that are treated with epigenetic erasers of the histone deacetylase inhibitor family.

1

Diffuse large B cell lymphoma (DLBCL) is the most common aggressive non‐Hodgkin lymphoma in adults, with seven to eight annual cases per 100 000 adults, a trend for higher incidence in men, and a peak at around 70 years of age. Drug combinations consisting of the CD20‐directed antibody rituximab, the DNA‐damaging drugs cyclophosphamide, doxorubicin, the microtubule poison vincristine and the glucocorticoid prednisolone are curative in nearly 70% of patients. This implies that over 30% of DLBCL patients cannot be cured, and refractory disease leads to poor overall survival of only 6 months.^1^

Novel drugs should be considered to provide salvage therapies for such patients. Histone deacetylases (HDACs) regulate the acetylation of histones and non‐histone proteins. A frequent dysregulation of HDACs in cancer cells has spurred an intense search on small molecules that inhibit them.^2^ This also applies to DLBCL cells in which an overexpression of HDAC1 ties in with worse prognosis.^3^ To date, five HDACs inhibitors (HDACi) have been approved by the Food and Drug Administration (FDA) USA and the FDA China for use in patients with cutaneous T cell lymphoma and multiple myeloma. These are active against all four zinc‐dependent HDACs (classes I, II and IV; pan‐HDACi; Figure 1A) or specifically target HDAC subtypes^2^, ^4^ (Figure 1B). A caveat of such epigenetic drugs is that existing markers for whether tumor cells are sensitive to HDACi are often not clinically validated, and HDACi have not been applied to stratified patient groups. To exploit the full potential of HDACi, unbiasedly collected evidence on drug sensitivity markers, developed for a specific tumor entity or subtype, is necessary.^5^

**FIGURE 1:**
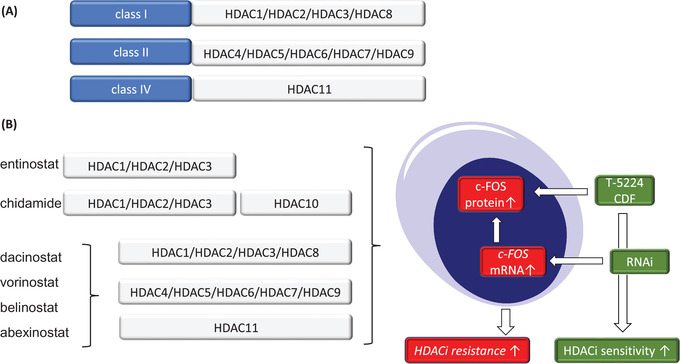
(A) Clinically relevant histone deacetylases inhibitors (HDACi) target the zinc‐dependent HDAC classes I, II and IV. These comprise the depicted HDACs, which are numbered according to their discovery. (B) Shown on the left are the HDACi that were used in the study that we comment on. These have broad or restricted activity against multiple HDACs. Less broad‐acting HDACi usually cause less side effects. The right side of the panel illustrates that HDACi modulate the transcription and expression of c‐FOS and how this affects diffuse large B cell lymphoma (DLBCL) cells. Inhibition of c‐FOS by RNAi or drugs sensitizes HDACi‐treated DLBCL cells to cell death

Wang, Wu, Xia and colleagues analyzed how epigenetic modifiers of the HDACi family affected DLBCL cells. These authors used 16 human DLBCL cell lines and treated them with the pan‐HDACi dacinostat (LAQ824) (Figure 1B). LAQ824 produced anti‐proliferative, apoptosis‐related effects in DLBCL cells, associated with a reduction of the anti‐apoptotic BCL2 protein, at least in some cell lines. Similar effects were found in DLBCL cells that were incubated with chidamide, which blocks HDAC1, HDAC2, HDAC3 and HDAC10^6^ (Figure 1B), suggesting that DLBCL cells require these HDACs to survive.

To characterize the dacinostat response, the authors conducted single‐cell RNA‐sequencing in activated B cell‐derived U2932 DLBCL cells that were treated with increasing concentrations of dacinostat. Considering a high apoptotic index after 24 h of dacinostat treatment, this approach was chosen to determine potential resistance networks in residual cells. Dimensionality reduction and clustering of single‐cell RNA‐sequencing data revealed seven distinct populations, of which three dominated in the high LAQ824 dose setting. Two of these clusters were characterized by increased expression of c‐FOS, being a core member of the activator‐protein‐1 (AP1) transcription factor family. Dimers of FOS (c‐FOS, FOS‐B, FRA‐1/FOSL1 and FRA‐2/FOSL2) and JUN (c‐JUN, JUN‐B, and JUN‐D) build the AP1 complex, which can drive tumor progression and treatment resistance.^7^ Consequently, the contribution of c‐FOS as a survival factor of residual cells was investigated. Here, most importantly, the sensitivity of DLBCL cells against HDACi was enhanced after knocking down c‐FOS by RNAi or the inhibition of the DNA binding capacity of c‐FOS with the compound T‐5224 and the more broadly acting agent difluorobenzocurcumin (CDF) (Figure 1B). It is promising that LAQ824 and CDF combined favorably against xenotransplanted U2932 DLBCL cells in mice.^8^ Further studies should carefully consider that an accumulation of c‐FOS was seen in vivo when U2932 DLBCL cells were exposed to LAQ824 and CDF. Such an undesired effect can promote rebound activation of c‐FOS when drug concentrations turn to the pharmacological nadir. Furthermore, high levels of c‐FOS were enriched in aggressive DLBCL cases,^8^ suggesting disease‐relevance of c‐FOS.

A clinically significant disadvantage of LAQ824 is that it caused cardiac problems in phase I studies, evidenced by dose‐related atrial fibrillation and QT prolongation. Therefore, LAQ824 was excluded from clinical use about 15 years ago.^9^ However, certain clinically valid implications of the here discussed study can be assumed because five other HDACi (chidamide, vorinostat, belinostat, abexinostat and entinostat, of which three are FDA‐approved) also induced c‐FOS^8^ (Figure 1B).

The mechanisms underlying the cytotoxic interaction between inhibition of c‐FOS and HDACi treatment were not elucidated, providing opportunities for additional research. Interestingly, a recent report points out that DLBCL cells with high activity of oxidative phosphorylation are less sensitive to the promising pan‐HDACi pracinostat and vorinostat.^10^ Reduced oxidative phosphorylation signatures were also noted by Wang, Wu, Xia and colleagues in LAQ824‐treated DLBCL cells. This likewise holds for a DNA repair hallmark. Congruent herewith and in agreement with previous studies,^5^ HDAC inhibition attenuated the activated, phosphorylated cell cycle regulator and DNA damage sensor kinase CHK2 and caused DNA replication stress/damage. Curiously, incubation of DLBCL cells with the DNA‐damaging drug doxorubicin induced c‐FOS more potently than LAQ824 did.^8^ It is tempting to speculate that apart from inducing histone phosphorylation and hyperacetylation, HDACi induce c‐FOS expression through DNA damage related pathways.

Although a limited number of patients were investigated, higher nuclear c‐FOS staining was detected in relapsed/refractory DLBCL, which might point to a more general role of c‐FOS in therapy resistance beyond HDACi. Furthermore, it is important to note that in the DLBCL in vivo model, growth was reduced by the HDACi and c‐FOS inhibitor combination therapy, but not completely.^8^ Whether, the third residual cell population emerging in the dacinostat high‐dose setting, characterized by low c‐FOS expression, contributes to the tumor outgrowth, and can be targeted, remains to be clarified.

Taken together, it appears that DLBCL cells call c‐FOS as pro‐survival transcription factor to the front upon epigenetic stress induction by HDACi. This notion suggests further development of AP1 inhibitors. It will be exciting to see whether other tumor entities also rely on c‐FOS for survival upon treatment with clinically applicable HDACi.

## CONFLICT OF INTEREST

The authors declare that there is no conflict of interest that could be perceived as prejudicing the impartiality of the research reported.
